# Light-Modulated Sunscreen Mechanism in the Retina of the Human Eye

**DOI:** 10.1021/acs.jpcb.1c01198

**Published:** 2021-05-26

**Authors:** Rafal Luchowski, Wojciech Grudzinski, Renata Welc, Maria Manuela Mendes Pinto, Alicja Sek, Jan Ostrowski, Lukasz Nierzwicki, Pawel Chodnicki, Milosz Wieczor, Karol Sowinski, Robert Rejdak, Anselm G. M. Juenemann, Grzegorz Teresinski, Jacek Czub, Wieslaw I. Gruszecki

**Affiliations:** † Department of Biophysics, Institute of Physics, Maria Curie-Sklodowska University, Pl. M. Curie-Sklodowskiej 1, 20-031 Lublin, Poland; ‡ Department of Interfacial Phenomena, Institute of Chemical Sciences, Faculty of Chemistry, Maria Curie-Sklodowska University, Pl. M. Curie-Sklodowskiej 3, 20-031 Lublin, Poland; § Department of General Ophthalmology, Medical University of Lublin, Chmielna 1, 20-079 Lublin, Poland; ∥ Department of Physical Chemistry, Gdansk University of Technology, Narutowicza 11/12, 80-233 Gdansk, Poland; ⊥ Viselle Augenzentrum Erlangen GmbH, 91052 Erlangen, Germany; # Department of Forensic Medicine, Medical University of Lublin, Jaczewskiego 8b, 20-090 Lublin, Poland

## Abstract

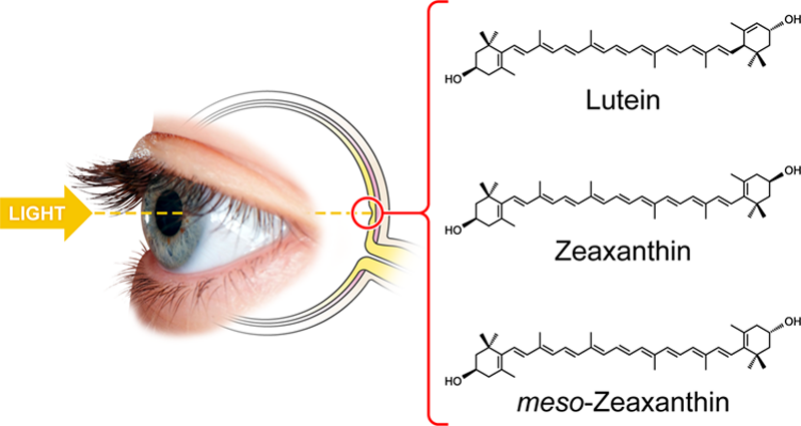

The functioning of the human eye in the extreme range of light intensity requires a combination of the high sensitivity of photoreceptors with their photostability. Here, we identify a regulatory mechanism based on dynamic modulation of light absorption by xanthophylls in the retina, realized by reorientation of pigment molecules induced by *trans*–*cis* photoisomerization. We explore this photochemically switchable system using chromatographic analysis coupled with microimaging based on fluorescence lifetime and Raman scattering, showing it at work in both isolated human retina and model lipid membranes. The molecular mechanism underlying xanthophyll reorientation is explained in terms of hydrophobic mismatch using molecular dynamics simulations. Overall, we show that xanthophylls in the human retina act as “molecular blinds”, opening and closing on a submillisecond timescale to dynamically control the intensity of light reaching the photoreceptors, thus enabling vision at a very low light intensity and protecting the retina from photodegradation when suddenly exposed to strong light.

## Introduction

**Figure 1:**
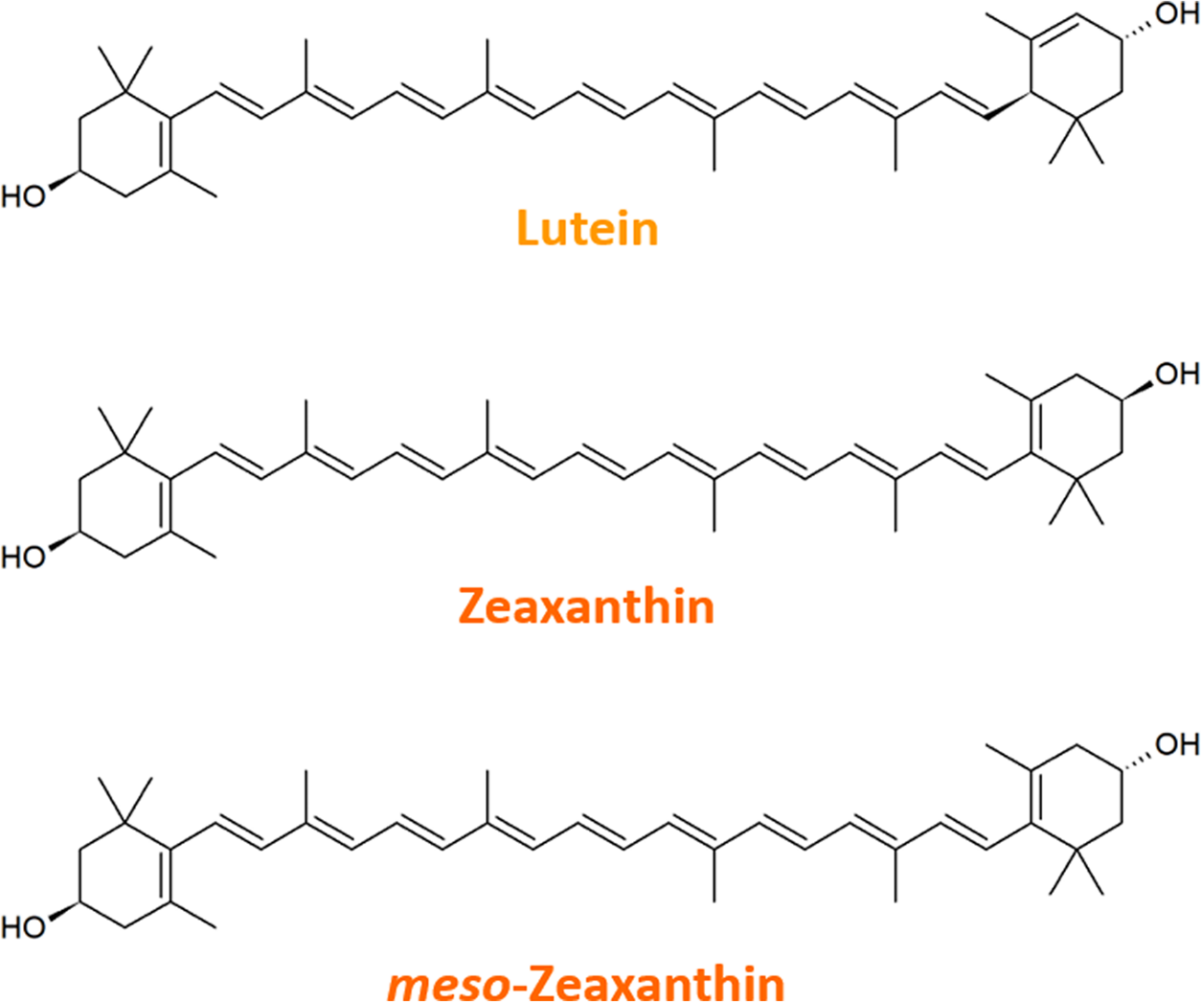
Chemical structures of the macular xanthophylls.

The xanthophylls lutein (Lut), zeaxanthin (Zea), and *meso*-zeaxanthin (*m*-Zea) (see Figure 1 for chemical structures) are indispensable constituents of the human retina, protecting photoreceptors against photodamage.^1−3^ The xanthophylls in the retina are present mostly in the yellow spot (*macula lutea*), the cone-rich region responsible for high-acuity central vision.^4^ A low level of the macular xanthophylls correlates with a high risk of age-related macular degeneration (AMD) that leads to irreversible vision loss.^5^ Photooxidative damage of biomolecules in the retina is recognized as one of the leading causes of AMD.^5^ Despite a general agreement regarding the protective activity of the macular xanthophylls against photodegradation,^2,6−8^ the specific molecular mechanisms involved in this activity in the retina are still not fully understood. In the present work, we address this problem both in the human retina and in the model membrane systems, providing a multiangle picture in which xanthophyll chromatography and molecular simulations complement the insights from spectroscopic imaging techniques based on time-resolved fluorescence and resonance Raman scattering. As a result, we present the operation of a number of molecular mechanisms that can be considered a manifestation of unknown regulatory activity in the human retina. The physiological role of this postulated regulatory mechanism is to dynamically adjust the number of photons reaching the photoreceptors in response to changes in light intensity.

## Methods

### Retina Preparation

Human eyes were collected and processed by the Eye Tissue Bank and the Department of Forensic Medicine of Medical University in Lublin in compliance with the Guidelines for Good Clinical Practice. Donor eyeballs were obtained and examined within 6–12 h post-mortem, in several cases after corneas had been removed for transplantation. All of the experiments were conducted in accordance with the approval of the Bioethics Commission affiliated with the Medical University in Lublin (decision No KE-0254/14/2017).

The retina samples were prepared from the eyeballs immediately after the section, under the dim light conditions. The vitreous, choroid, sclera, and other structures were separated leaving the intact retina with a yellow macula spot distinctly visible. The samples in phosphate-buffered saline (PBS) were then transferred to quartz slides adequate for fluorescence and Raman imaging and immediately subjected to examination. To Raman imaging, the retina samples were transferred to 80% glycerol solution, cooled down to −30 °C, and scanned using a temperature-controlled stage THMS600 (Lincam, U.K.) with an additional thermocouple to monitor the temperature directly in the sample. In total, the retina samples prepared from 69 eyeballs from 35 donors were examined.

### Materials

1,2-Dipalmitoyl-*sn*-glycero-3-phosphocholine (DPPC) and 1-palmitoyl-2-oleoyl-*sn*-glycero-3-phosphocholine (POPC) were purchased from Avanti Polar Lipids, Inc. Crystalline xanthophylls (all-*trans*)-Lutein [(3*R*,3′*R*,6′*R*)-β,ε-carotene-3,3′-diol] and (all-*trans*)-Zeaxanthin [(3*R*,3′*R*)-β,β-carotene-3,3′-diol] were purchased from Extrasynthese. (All-*trans*) *meso*-Zeaxanthin [(3*R*,3′*S*)-β,β-carotene-3,3′-diol] was obtained from U.S. Pharmacopeia. Methanol and methyl *tert*-butyl ether were purchased from POCH (Poland) and were of chromatographic quality.

### Xanthophyll Purification and Analysis

To remove possible degradation products, directly before use, xanthophylls were purified chromatographically with the application of a Shimadzu LC-20AD system equipped with an SPD-M20A diode-array detector and with a C-30 coated, phase-reversed column (YMC GmbH, Germany), internal diameter 4.6 mm, length 250 mm, and particle size 5 μm. A mixture of methanol and methyl *tert*-butyl ether (95:5, v/v) was used as a mobile phase and the flow rate was 1 mL/min. The detailed methods of the xanthophyll isomerization and identification were described previously.^9^

A chromatographic column has been selected and separation conditions optimized to ensure separation of geometric molecular configuration forms of carotenoids. The xanthophyll analysis of the human retinas was performed on a Nexera LC-40 (Shimadzu, Kyoto, Japan) ultrahigh-performance liquid chromatography (UHPLC) system equipped with a diode-array detector SPD-M40, an FCV-0607H high-pressure flow-line selection valve, a CTO-40C column oven, a SIL-40C XR autosampler, and an LC-30D XR pump. The flow rate was 0.2 mL/min. The isocratic separation of carotenoids was carried out on a Stability 100 C30 column, 150 mm × 3 mm internal diameter, pore size 100 Å, particle size 3 μm (Dr. Maisch, Ammerbuch, Germany), equipped with a ReproSil Universal RP 3 mm × 4 mm id., particle size 5 μm (Dr. Maisch, Ammerbuch, Germany). The autosampler was set to 4 °C, and the column was thermostated at 30 °C. The mobile phase consisted of methanol and methyl *tert*-butyl ether (95:5 v/v). The injection volume was 5 μL, and the runtime 35 min. The peak spectra were scanned from 300 to 800 nm. Isolation of xanthophylls was carried out based on ref (10) with slight modifications. In short, after dissecting fresh eyeballs, the prepared central retina (1 cm diameter disk) containing the yellow spot was transferred to a glass tissue homogenizer and 2 mL of acetone was used for extraction of carotenoids. Acetone extracts were filtered through 0.2 μm syringe filters and dried under pure argon in glass vials. Dried samples were redissolved by briefly swirling cold (−20 °C) acetone and transferred to a clean vial and immediately injected on the HPLC. All preparatory activities were carried out in a dark room with diffused dim light.

### Giant Unilamellar Vesicles (GUV) Preparation

Giant unilamellar vesicles (GUV) were prepared of DPPC with xanthophylls according to the method described previously.^11^ GUV were formed via the electroformation method at 0.5 mol % xanthophyll concentration with respect to DPPC. Directly before liposomes preparation Lut, Zea and *m*-Zea were repurified using the HPLC technique and then the proper amount of investigated carotenoid has been added to an ethanolic solution of lipid. The obtained mixture was deposited at two platinum electrodes fixed in the Teflon holder at a distance of 3 mm, incubated in vacuum for 1 h (to remove organic solvent residues) and next placed in a cuvette containing a buffer solution (1.4 mL, 20 mM Tricine, 10 mM KCl, pH 7.6). Electric connections were then attached to the AC field supply (DF 1641A) and the electroformation process was started. It was carried out over 2 h with 10 Hz frequency and 3 V voltage (peak-to-peak). The temperature was stabilized at 45 °C.

### Fluorescence Lifetime Imaging Microscopy (FLIM) Measurements

Microscale imaging based on fluorescence lifetime analysis was carried out with the application of a fluorescence lifetime imaging microscopy (FLIM) system. FLIM measurements were performed using a two-channel confocal MicroTime 200 (PicoQuant, Germany) system connected to an inverted microscope Olympus IX71. A confocal pinhole of 50 μm in diameter was used. Results of measurements were analyzed with the application of SymPhoTime 64 software. Fluorescence in the samples was excited with a 470 nm solid-state pulse laser (68 ps full width at half-maximum) with an increasing repetition frequency in the range of 0.2 to 32 MHz corresponding to increasing laser power pointed on the figures. Fluorescence photons were collected with a 100× oil objective (NA 1.4, Olympus UPlanSApo) in the case of liposome analysis and an Olympus objective with 60× magnification, 1.4 numerical aperture, and immersion in the form of silicone oil in the case of imaging the retina samples. The fluorescence signal was split by the polarizing cube and observed by two orthogonally polarized analyzers. In the case of imaging of the retina samples temperature was stabilized at 36.6 °C. For the liposome imaging, a constant temperature of 45 °C, above the main phase transition of membranes formed with DPPC, was maintained. Fluorescence was measured in a time-tagged time-resolved mode and was filtered by two dichroic filters: Notch 470 and band-pass 550/88 (both from Semrock, Inc.) focused on carotenoid emission observation. The detection was accomplished with two identical single-photon avalanche diode detectors (type tau-SPAD from Picoquant GmbH) characterized with timing resolution down to 350 ps or silicon avalanche photodiodes type SPCM-AQRH-TR Excelitas with timing resolution down to 250 ps.

The lifetime analyses were performed in SymPhoTime 64 software based on deconvolution of fluorescence intensity decays with instrument response functions. In the case of the retina samples, the number of lifetime components was three and for most cases four, depending on the imaging area and depth of the retina. The biological samples required certain freedom in fitting the emission decay curves. The fluorescence lifetime components τ_i_ used for deconvolution changed within the ranges specified as below: 80 ± 5 ps, 0.3 ± 0.05, 1.6 ± 0.4, and 6 ± 2 ns. The intensity-averaged fluorescence lifetime was calculated according to the formula
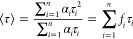
where τ is the decay lifetime, *n* is the number of lifetime components, α_*i*_ is the amplitude of the *i*th decay component , and *f*_*i*_ is the amplitude of the component, representing intensity-averaged fluorescence lifetime.

Fluorescence lifetime images were collected for 80 μm × 80 μm areas with 400 × 400 pixel resolution. Signals from both the detectors were used to calculate the anisotropy in each pixel according to the formula presented below
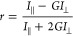
where *I*_∥_ and *I*_⊥_ are the fluorescence intensity signals for parallel and perpendicular emissions relative to the polarization of the excitation light, respectively, and *G* is an apparatus factor determined before each retinal measurement on the basis of long-lifetime fluorophore in the used spectral region (usually close to 1).

Fluorescence emission spectra of retinal xanthophylls were recorded from single axons with the same microscopy system upgraded with the spectrograph SR-163 equipped with a Newton 970 EMCCD camera from Andor Technology.

The time course of xanthophyll fluorescence in a single axon was measured with the MicroTime 200 confocal system (PicoQuant). The experimental arrangement was the same as described above. Confocal volume illuminated by the laser was determined based on scanning of fluorescence beads (30 nm in diameter) in *x*–*y* and *x*–*z* directions and calculated as an ellipsoid of revolution. An effective volume of an ellipsoid is (π/2)^3/2^ω_o_^2^*z*_o_, where ω_o_ it’s the lateral and *z*_o_ it’s the axial radius. Taking into account ω_o_ = 312 nm and *z*_o_ = 940 nm microscope operated with the effective confocal volume of 0.18 fl. We collected data points every 10 μs for 180 s and smoothed the data by binning 10^5^ points.

### Raman Imaging of the Retina

Raman imaging was carried out using an inVia confocal Raman microscope (Renishaw, U.K.) with an argon laser (Stellar-REN, Modu-Laser) operating at 488 nm, equipped with 20× long distance objective (Olympus Plan N NA = 0.25). Optical images of the human retina were obtained and elaborated with WiRE 4.4 software (Renishaw, U.K.). Based on the optical image from the area of approx. 10 mm × 10 mm, a 4 mm × 4 mm area was selected, covering the yellow spot and imaged with a spatial resolution of 5 μm (pixel size 5 μm × 5 μm). For this study, all of the images were recorded with a light power of 500 μW or attenuated to lower laser powers if necessary (indicated). At each point of the Raman image map, the spectra were recorded with about 1 cm^–1^ spectral resolution (2400 lines/mm grating) in the spectral region 800–1800 cm^–1^ using EMCCD detection camera Newton 970 (Andor, U.K.). Images were acquired with the use of the Renishaw WiRE 4.4 system at high-resolution mapping mode (HR maps). The acquisition time for a single spectrum was 0.1 s. All spectra were preprocessed by cosmic ray removing and baseline correction using WiRE 4.4 software from Renishaw, U.K. For comparison of dark-adapted and illuminated retina samples spectra were recorded in the course of microscopic imaging, from the neighboring parts of the *macula lutea*, one incubated in darkness (dark) and the other exposed for 5 min to white light (white diode illuminator) at the intensity of 750 μmol photons/m^2^s (light). The sectors of the retina were incubated in the dark and exposed to light at 36.6 °C, and then the temperature was lowered to −30 °C at which the spectra were recorded.

### Raman Imaging of GUV

Raman imaging was carried out using an inVia confocal Raman microscope system (Renishaw, U.K.) with an argon laser (Stellar-REN, Modu-Laser) operating at 488 nm, equipped with 60× water immersed objective (Olympus PlanApo NA = 1.2). Based on optical images of xanthophyll-containing GUV, elaborated with WiRE 4.4 software (Renishaw, U.K.), areas for Raman scanning were selected and mapped at high-resolution mapping mode with 0.5 μm spatial resolution. Images were recorded at a temperature of 45 °C with light intensity in the range between 6.0 μW (0.5% of the laser power) and 97.0 μW (10%), as measured in the sample compartment. At each point of the Raman image, spectra were recorded with about 1 cm^–1^ spectral resolution (2400 lines/mm grating) in the spectral region from 445 to 1885 cm^–1^ and 0.05 s exposure time. EMCCD Newton 970 camera (Andor Technology, U.K.) as a detector was used. All spectra were preprocessed by cosmic ray removing, noise filtering, and baseline correction using Renishaw software. At least 10 giant unilamellar vesicles with Lut, Zea and *m*-Zea were imaged and analyzed. Representative images are presented in the paper.

Principal component analysis (PCA) was used as the first step in Raman image analysis to identify the number of different xanthophyll spectra and assign them to specific stereochemical forms both in the GUV and retina samples. The analysis was then performed based on direct classical least squares (DCLS) component analysis based on predefined component reference spectra to map the distributions of different spectral forms of xanthophylls. The PCA and DCLS protocols are part of the WiRE (v. 5.2) software of the Renishaw inVia microscope system.

## Computational Methods

### Hydrophobic Lengths of Xanthophylls

The hydrophobic lengths of xanthophyll molecules D_O–O_ were calculated as the distance between their hydroxyl oxygen atoms. The kinetically stable rotamers of the β-ionone and ϵ-ionone rings were determined by relaxed potential energy scans of the dihedral angle C5-C6-C7-C8 (see Table S1) with Gaussian09^12^ using the MN12-SX^13^ functional and 6-31G(d) basis set.

### Molecular Dynamics (MD) Simulations

Simulation systems consisted of a single Zea molecule (either all-*trans*, 9-*cis* or 13-*cis* isomer) embedded in a lipid bilayer composed of 146 DPPC molecules, solvated with 5508 water molecules with 20 potassium and 20 chloride ions to provide physiological ionic strength. Additionally, each of the three Zea isomers was simulated in a lipid bilayer consisting of 90 DSPC, 84 SDPE and 26 SDPS molecules, representative of the lipid composition of the retinal cell membranes,^14^ solvated with 6896 water molecules with 42 potassium and 16 chloride ions. All of the systems were built using the CHARMM Membrane Builder.^15^

All simulations were carried out using NAMD 2.10,^16^ using CHARMM36 force field^17^ and the TIP3P water model. The NPT ensemble was used to perform simulations with the temperature maintained at 320 K by Langevin dynamics and pressure maintained at 1 bar with the Langevin piston method.^18^ Long-range electrostatic interactions were calculated with the Particle Mesh Ewald algorithm with a real-space cutoff of 10 Å. Van der Waals interactions were described using Lennard-Jones potential with a 12 Å cutoff using a switching radius of 10 Å. The velocity Verlet algorithm was used to integrate equations of motion with a time step of 2 fs. All covalent bonds involving hydrogen were constrained with the SHAKE algorithm.

Simulated systems were energy-minimized and subsequently equilibrated in six steps, using the standard CHARMM Membrane Builder protocol. Then, umbrella sampling (US) was used to obtain the free energy profiles governing the orientation of the Zea molecules with respect to the bilayer normal. As the reaction coordinate for US, we used the distance between the centers of mass of the carbon atoms of each half of the polyene chain (C8–C15) projected on the bilayer normal. The initial configurations for the US simulations were obtained from 40 ns steered-MD simulation, during which Zea molecules were forced to change their orientation from perpendicular to parallel using a time-dependent harmonic potential with a spring constant of 10 kcal mol^–1^ Å^–2^. In all cases, 10 evenly spaced US windows separated by 1.2 Å were used to span the reaction coordinate. Each window was subject to a harmonic bias potential of 3 kcal mol^–1^ Å^–2^ to efficiently sample the entire range of the reaction coordinate. In each of these windows, the system was simulated for 500 ns and the resulting distributions of the reaction coordinate were post-processed using weighted histogram analysis method (WHAM)^19^ to calculate the corresponding free energy profiles. Uncertainties of the free energy profiles were estimated by means of Monte Carlo bootstrap method taking time series correlations into account. Finally, the obtained free energy profiles were mapped to the angle between the xanthophyll polyene chain and the membrane normal (tilt angle, θ), using one-to-one mapping.

For each Zea isomer (all-*trans*, 9-*cis*, and 13-*cis*), two sets of 10 maximally independent structures were extracted from the free energy simulations, one corresponding to the vertical and one corresponding to the horizontal orientation of the molecule in the bilayer. Then, Gaussian was used to perform time-dependent density functional theory (TD-DFT) calculations on each structure in two variants, either (a) using the ONIOM approach, with a shell of neighboring molecules (∼1800 atoms on average), or (b) in vacuum. The MN12L functional was used along with the cc-pVTZ basis set, and six excited states were calculated. From each calculation, the transition dipole moment corresponding to the highest oscillator strength was selected, and the angle between the transition dipole moment and the main inertia axis of the molecule was calculated, always assuming the same orientation between the vectors (i.e., choosing values in the 0–90° range).

## Results

### Photochromic-like Activity of Xanthophylls in the Retina

**Figure 2:**
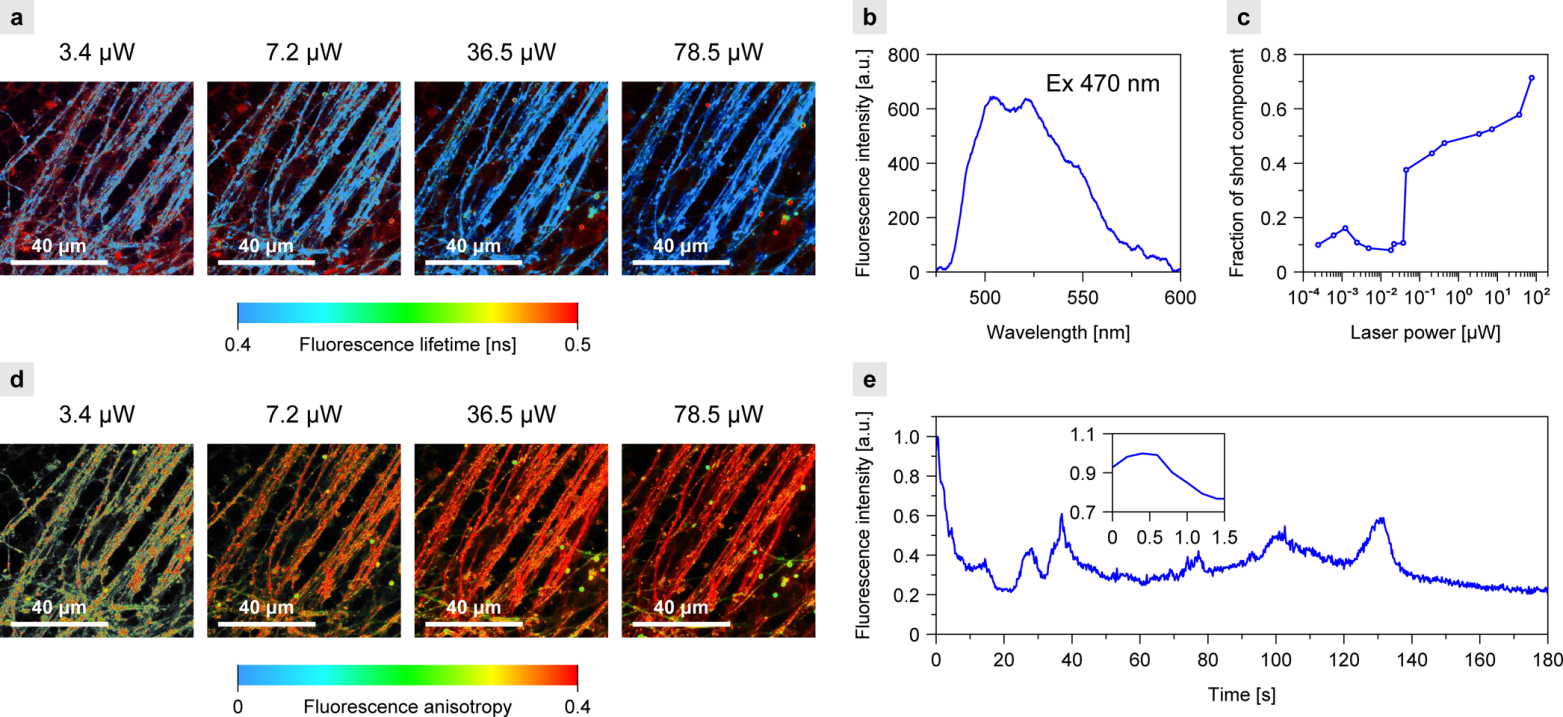
Imaging and fluorescence analysis of the human retina. (a) FLIM imaging with increasing laser power. (b) Fluorescence emission spectrum of a single axon. (c) Laser power dependency of the fraction of the short-lifetime component in the experiment presented in (a). (d) Fluorescence anisotropy images corresponding to the FLIM images in (a). (e) Time course of xanthophyll fluorescence changes in a single axon at laser power 1.4 μW. Fluorescence detection started automatically by the system 1 s after the onset of laser light. The maximum signal normalized to 1. The retina from a healthy 18-year-old female donor (a–d) and a healthy 39-year-old male donor (e).

Figure 2a presents the fluorescence lifetime imaging microscopy (FLIM, see also Figure S1a) images of axons in the outer plexiform layer of the fovea region of the retina, recorded with increasing laser power. Anatomical structures appearing in blue in the color-coded FLIM images represent xanthophyll-rich regions characterized by relatively short fluorescence lifetimes (timescale of picoseconds, Figure S1c).^20^ This assignment is confirmed by the fluorescence spectra recorded from single axons (Figure 2b), typical for the emission of polyenes with the conjugated double-bond system *N* = 10 and 11.^21^ To our surprise, we found that increasing probing light intensities yielded drastically different relative amplitudes of the short-lifetime component representing xanthophylls (Figures 2a,c and S1d). The total number of photons detected in these experiments increased linearly with increasing probing light intensity, ruling out the possibility of pigment photobleaching while imaging (Figure S1e). Hence, given a constant fluorescence quantum yield, these light-intensity-dependent changes in xanthophyll fluorescence can only be attributed to changes in their light absorption. This means that low light intensity (below 10^–2^ μW), induces a decrease in light absorption by xanthophylls in axons, while an opposite effect can be detected at strong light (∼3 × 10^–2^ μW, Figures 2c and S1d). Importantly, the effect of light is reversible in darkness (Figure S2). The increase in the short-lifetime fraction in axons is accompanied by the increase in fluorescence anisotropy (Figures 2d and S1b), indicative of light-induced remodeling of the xanthophyll-comprising systems. One of the most plausible explanations of this effect is that chromophores reorient toward the plane of the retina such that a greater number of molecular dipole transitions were potentially collinear with the electric vector of probing laser light (see Figures 2d and S1b). It has to be noted that these light-driven effects occur on a submillisecond timescale as the scan time of a single pixel was 0.6 × 10^–3^ s. Interestingly, prolonged illumination of a single axon causes oscillation-like changes in xanthophyll fluorescence that follow the initial increase (Figure 2e). The nature and possible mechanism of this observation will be discussed below.

### Light-Induced Reorientation of Xanthophylls in Lipid Membranes

**Figure 3:**
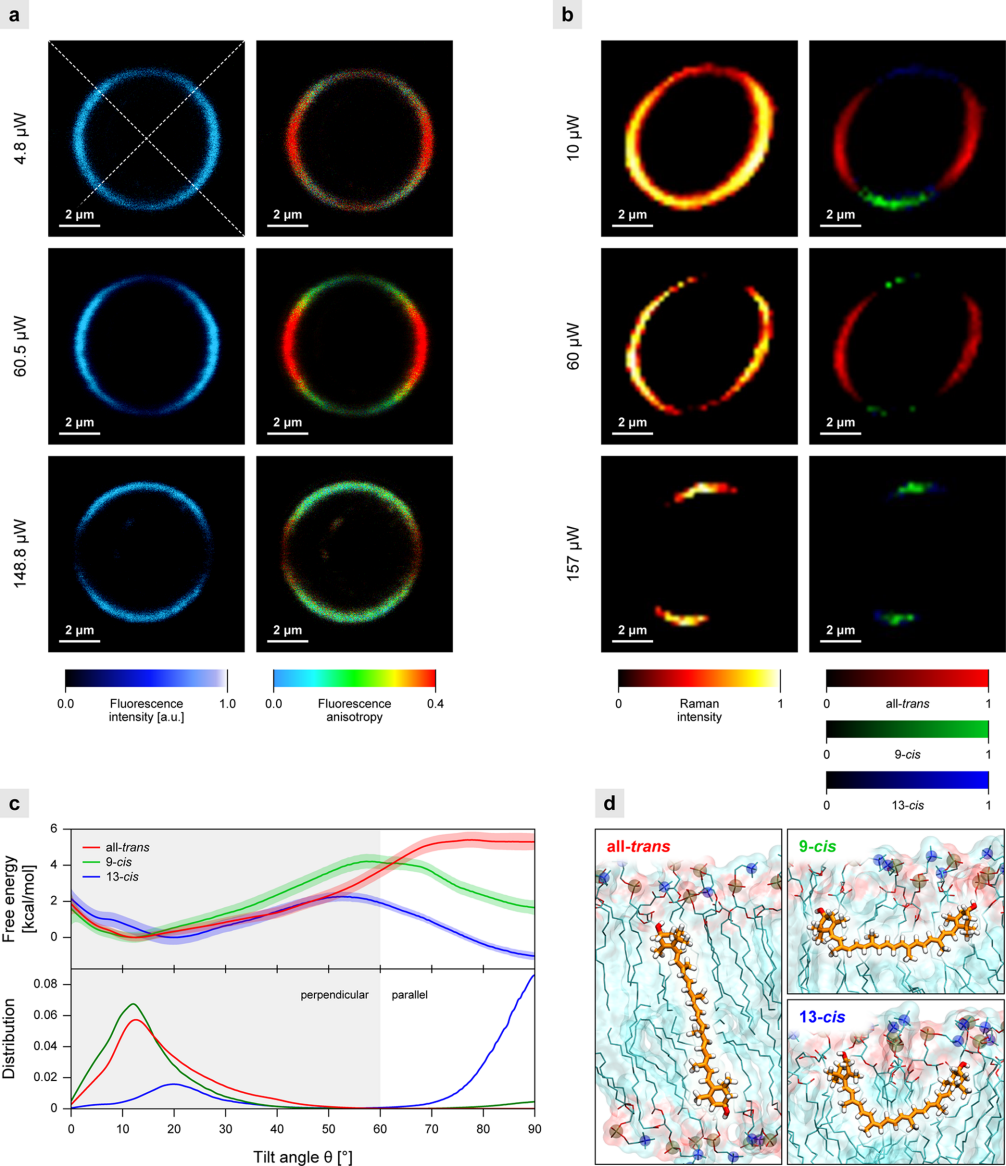
Experimental and computational analyses of xanthophylls in model membranes. Images of the equatorial cross section of the lipid vesicle containing *m*-Zea (a) or Zea (b) recorded with increasing laser power. Imaging is based on fluorescence intensity and anisotropy (a) or Raman scattering (b). On the right-hand side of (b), results of the component analysis are shown. Molecular configurations all-*trans*, 9-*cis*, and 13-*cis* were identified (the component spectra are shown in Figure S5). (c) Free energy profiles (top) and the corresponding probability densities (bottom) for the tilt angle between the polyene chain and the membrane normal (see Figure S7a) for Zea all-*trans* and its 9-*cis* and 13-*cis* isomers in the DPPC bilayer. (d) Representative structures for the perpendicular orientation of all-*trans* Zea (left) and the horizontal orientations of its two *cis* isomers (right) in the DPPC membrane.

**Figure 4:**
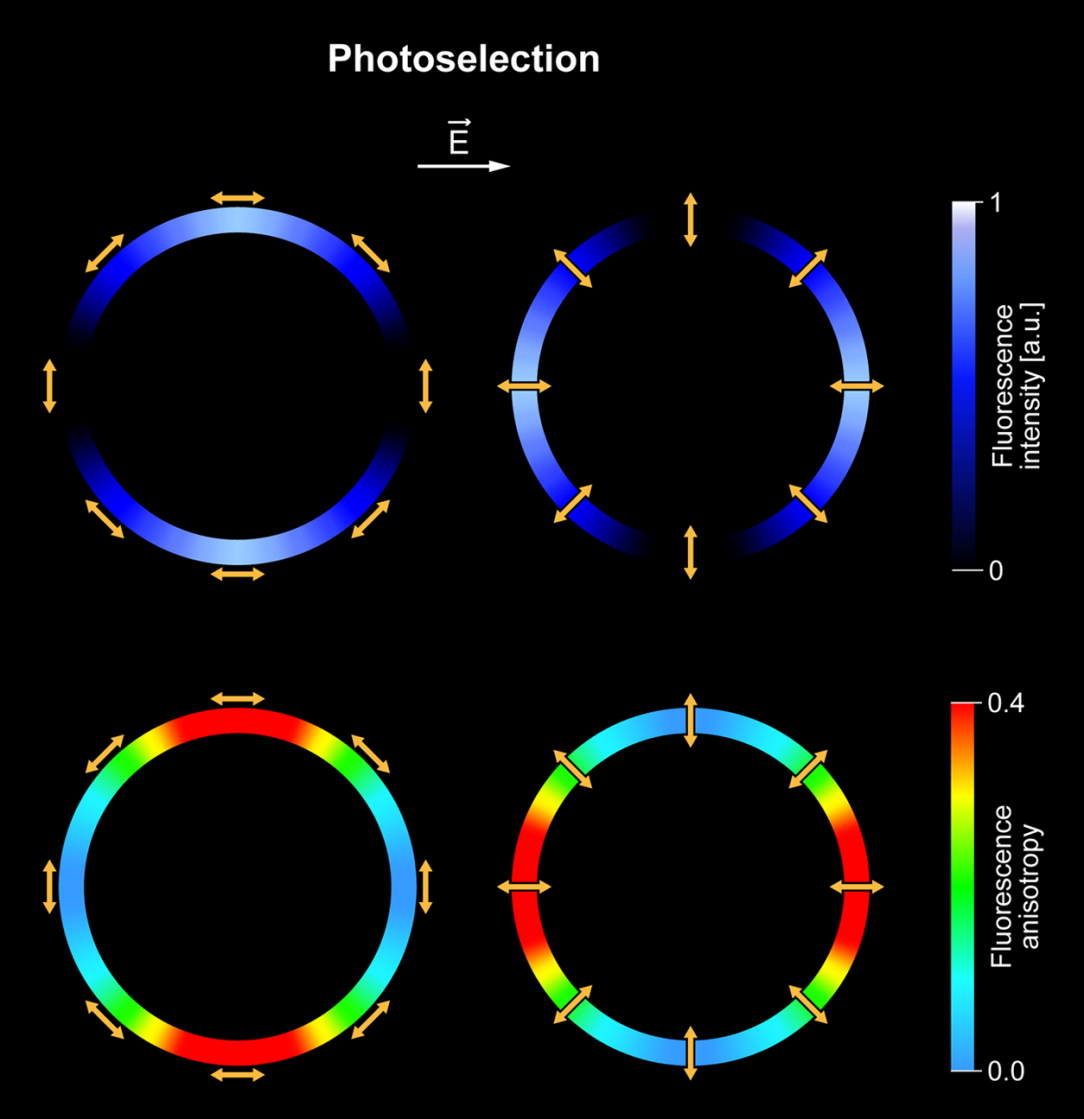
Schematic representation of the idea of photoselection. Color rings represent the equatorial cross sections of the spherical lipid vesicles: imaged by means of fluorescence (top) or fluorescence anisotropy (bottom). Linear fluorophores represented by yellow arrows are bound to the membranes and oriented parallel (left) or perpendicular (right) with respect to the membrane plane of a lipid vesicle. The highest fluorescence signals and fluorescence anisotropy values are observed in the cases when the electric vector of the laser scanning light (*E⃗*) is parallel to the transition dipole of the light-absorbing molecules. According to this effect, linear chromophores oriented vertically to the membrane plane give rise to high fluorescence intensity and anisotropy levels on the left- and right-hand sides of the cross section of the lipid vesicle and the chromophores oriented horizontally to the membrane plane give rise to the high signals in the top and bottom parts of the liposome.

The fact that light-induced changes in xanthophyll fluorescence are accompanied by changes in fluorescence anisotropy suggests the possibility of light-induced reorganization of xanthophyll-lipid membranes. To address this problem, we turned to model system studies. Figure 3a presents the FLIM images of a single giant unilamellar vesicle (GUV) containing *m*-Zea. The dominant vertical orientation of chromophores within the membrane can be concluded based on photoselection (see the model in Figure 4).^11^ This is due to the fact that higher fluorescence intensity in the image of the equatorial section of a single vesicle can be observed in the left and right sectors (see the axes drawn with white dotted line).^11^ The increase in laser power causes a clear redistribution of the fluorescence intensity so that higher intensity can be observed in the upper and lower sectors of the liposomes (Figures 3a and S3). Such an effect is indicative of the light-induced reorientation of xanthophylls to a horizontal position (Figure 4). The same effect can also be observed in the Raman imaging of a single GUV (Figures 3b and S4).^11^ Importantly, the analysis of the Raman spectra reveals that the light-induced reorientation of xanthophylls in the membranes is associated with their molecular reconfiguration. The component analysis shows the presence of three spectral forms assigned to all-*trans*, 9-*cis*, and 13-*cis* configurations (Figures 3b, S4, and S5), even though the chromatographically pure all-*trans* xanthophylls were originally incorporated into the membranes. The most likely explanation of this effect is a xanthophyll photoisomerization over the course of GUV imaging.^9^ Importantly, according to the analysis of distribution of different geometric forms of xanthophylls, a molecular axis orientation within the membrane depends critically on its configuration: all-*trans* is perpendicular while 9-*cis* and 13-*cis* are identified as parallel to the membrane plane (Figures 3b and S4). As can be expected, the initial incorporation of the *cis* forms of xanthophylls results in the dominant horizontal orientation of their molecules, even at low probing light intensities (Figure S6). Light-induced conversion of *cis* xanthophylls to the all-*trans* form (Figure S6) implies that such photoisomerization can account for restoring the pool of the all-*trans* xanthophylls, observed also in the retina (Figure S2).

**Figure 5:**
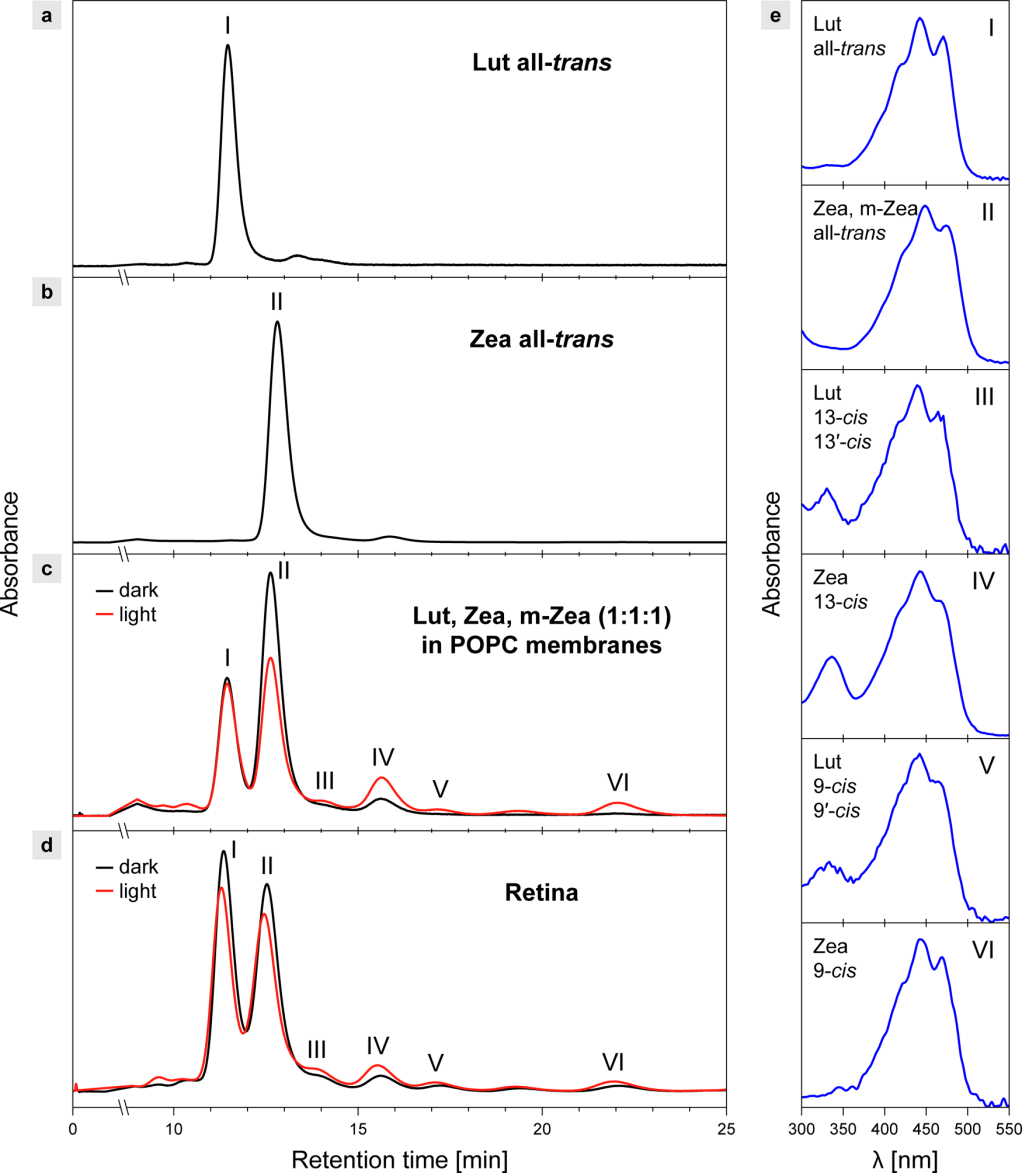
HPLC analysis of macular xanthophylls. (a) Chromatogram of a standard of all-*trans* Lut. (b) Chromatogram of a standard of all-*trans* Zea. (c) Chromatograms of a mixture of Lut:Zea:*m*-Zea (1:1:1, by mole) incorporated into GUV membranes formed with POPC. The concentration of each xanthophyll 0.5 mol % with respect to lipid. (d) Chromatograms of the xanthophyll extracts from the central retina prepared from the eyeballs of the healthy 52-year-old male donor (the left eye, dark-adapted, the right eye, illuminated). (e) Absorption spectra from the diode-array detection systems representing the elution bands I–VI numbered in (a–d). The spectra were acquired in the course of the experiment presented in (c). The assignment based on the reference spectra.^9^ (c, d) Analyses of the samples incubated for 30 min in the dark at 36.6 °C (dark) or exposed afterward for white light with 750 μmol photons m^–2^ s^–1^ for 5 min (c) or illuminated by a single flash (0.005 s) of a photographic camera (Canon EOS 600D) from a distance of 30 cm. The flash lamp is characterized by a guide number 13 (ISO100, meters). The retina samples were kept in a physiological salt solution during dark incubation and light exposure. Chromatograms are based on the absorbance level at 442 nm. Presented chromatograms are area-normalized.

The interpretation regarding light-induced xanthophyll reorientation triggered by *trans*–*cis* photoisomerization has direct support from the results of chromatographic analysis. We detected the light-driven xanthophyll *trans*–*cis* isomerization in both model lipid membranes and the human retina in the HPLC analyses (Figure 5). Interestingly, in all cases, xanthophylls in *cis* molecular configurations were detected even in the dark-adapted samples, and even though only all-*trans* forms were incorporated into the model lipid membranes (Figure 5c). Most likely, an equilibrium between the *trans* and *cis* forms is reached spontaneously, at a level dependent on the actual environment and thermodynamic conditions, owing to the relatively low energy barrier for both the *trans*-to-*cis* and *cis*-to-*trans* interconversions.^22^

### Molecular Mechanism Underlying the Xanthophyll Reorientation in the Membranes

To verify and extend the conclusions drawn from the fluorescence and Raman imaging, we examined to what extent the *trans*–*cis* isomerization of xanthophyll molecules affects their orientation in lipid bilayers. With this purpose, we used molecular dynamics (MD) to compute the free energy profile for the tilt angle θ between the bilayer normal and the polyene chain of a single all-*trans*, 9-*cis*, and 13-*cis* Zea molecule embedded in a DPPC membrane. As shown in Figure 3c, all-*trans* Zea occurs almost exclusively in the vertical orientation with an average tilt angle of 23.6° and a 5 kcal/mol preference over the horizontal orientation. When vertically oriented, all-*trans* Zea spans the membrane with the two hydroxyl groups interacting with the opposite membrane surfaces (Figure 3d). However, isomerization to 9-*cis* Zea increases the fraction of horizontally oriented molecules, with an average tilt angle of 84.3°, to almost 20%. An even more pronounced tendency to align with the membrane plane is observed in the case of 13-*cis* Zea, for which over 80% of the molecules are horizontally oriented. In this orientation, Zea resides next to the membrane-water interface, enabling the hydroxyl groups to interact favorably with the polar region of a single monolayer (Figure 3d). Using a TD-DFT excited-state analysis, we confirmed that for all isomers the transition dipole moment aligns almost perfectly with the main inertia axis of the molecule (Figure S7b), allowing us to use one as a good proxy for the other even in case of a considerably nonlinear molecule such as 13-*cis* Zea. Overall, these findings further support the notion that photoisomerization of all-*trans* xanthophylls to the 9- and, particularly, 13-*cis* molecular configuration switches their orientation in the membrane from vertical to mostly horizontal.

The molecular basis of this rearrangement can be readily explained in terms of the hydrophobic mismatch between the thickness of the membrane hydrophobic core and the length of Zea. The *trans*–*cis* isomerization shortens the hydrophobic length of Zea, defined as the distance between its polar hydroxyl groups (see Table S1). Although the Zea *trans* isomer is still long enough to span across the 3.09-nm-thick hydrophobic core of the DPPC membrane (Figure S7c), *cis* isomers are too short to simultaneously interact with polar regions of both membrane leaflets. Furthermore, the *cis* configuration of Zea promotes the hydroxyl groups to be on the same side of the molecule (Figure S7d–f), which facilitates hydrogen bonding with the polar membrane surface and thereby provides additional stabilization of the parallel oriented 9- and 13-*cis* isomers.

With additional MD simulations of a lipid bilayer corresponding to the average composition of the retinal liquid-disordered membrane phase,^14^ we evaluated its thickness to 3.16 nm (Figure S7c) in very good agreement with the hydrophobic thickness of the human rhodopsin receptors (3.18 ± 0.11 nm, PDB 4ZWJ).^23^ This implies that one should observe a considerable fraction of the horizontally oriented *cis* isomers also in the retina. Indeed, the free energy profiles computed for the 9-*cis* and 13-*cis* Zea isomers (Figure S7e) show that 2 and 45% of the molecules, respectively, adopt the horizontal orientation in the model retinal membrane.

### Photoconversion of Xanthophylls in the Retina

**Figure 6:**
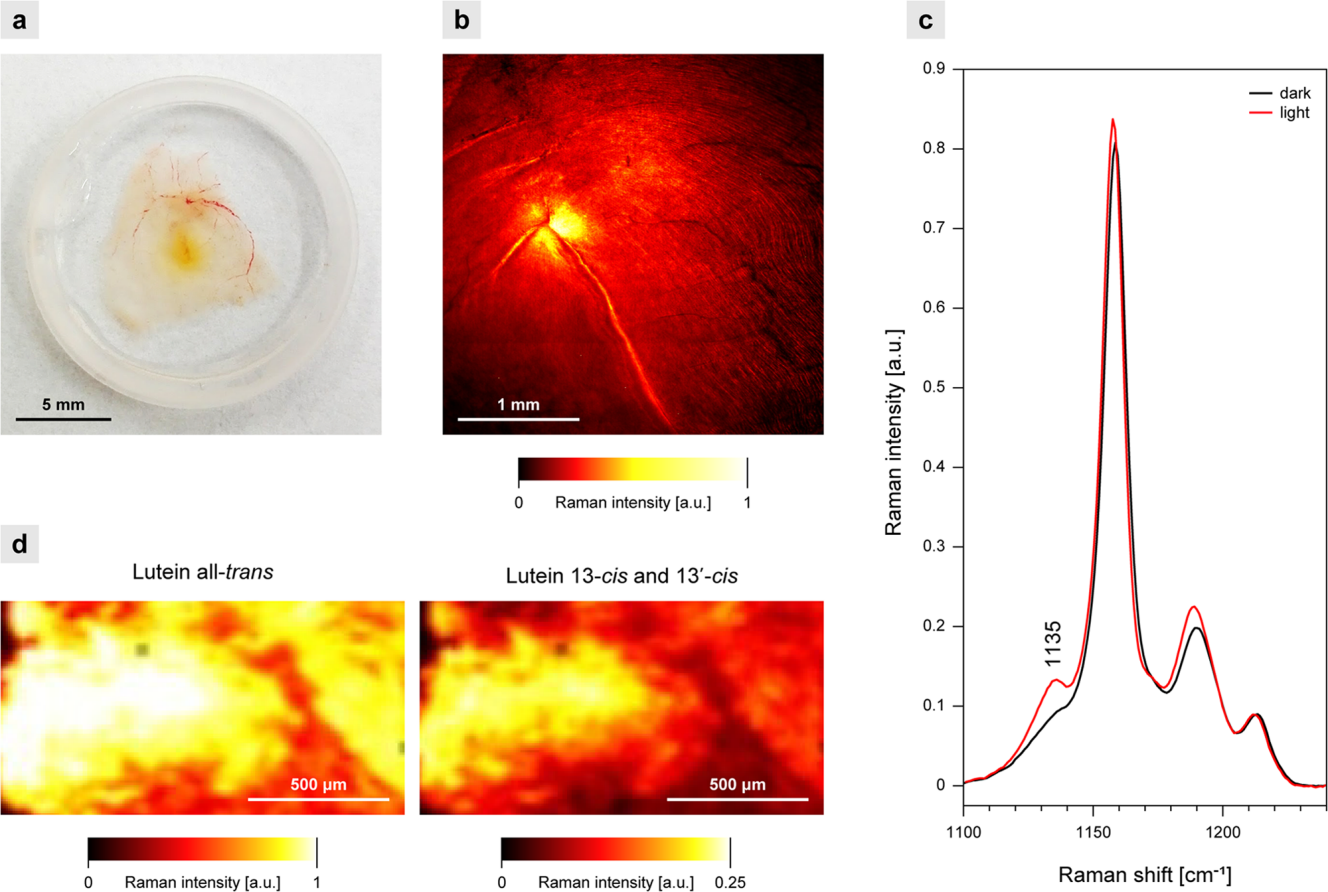
Raman analysis of the human retina. Experiments with a 488 nm laser. (a) Photo of a preparation. (b) Raman image based on the integration of the ν_1_ band (1500–1550 cm^–1^). (c) Raman spectra in the ν_2_ region, recorded from the neighboring parts of the macula: dark-adapted (dark) and exposed for 5 min to white light (light). Spectra represent the arithmetic mean from 20 spectra recorded at different positions. The spectra are normalized in the maximum of the ν_1_ band. (d) Distribution of the molecular configuration forms of Lut in the central retina exposed to light. Note different scales in the images representing the all-*trans* and 13-*cis* and 13′-*cis* isomers. In the case of Lut all-*trans*, the intensity is overestimated owing to the contribution of Zea and *m*-Zea to the resonance Raman spectra. The retina samples from a healthy 79-year-old female donor (a), a healthy 34-year-old female donor (b), and a healthy 18-year-old female donor (c, d).

The results of the chromatographic analysis show that a certain fraction of macular xanthophylls appears in the 13-*cis* molecular configuration, even in the dark-adapted retina (∼10% in the case of Zea and *m*-Zea), and that this fraction increases upon light exposure (Figure 5d). To visualize the distribution of *cis* molecular configuration forms of xanthophylls in the retina, we examined retina samples with the application of Raman imaging (Figure 6). In principle, two different resonance Raman spectra can be resolved while scanning the retina, originating from xanthophylls with two different lengths of the conjugated double-bond system: *N* = 10, assigned to Lut, and *N* = 11, assigned to Zea and *m*-Zea.^24^ Application of a 488 nm laser enables imaging of the retina based on the localization of all macular xanthophylls, although a particular resonance with Lut can be expected owing to matching the 0–0 electronic transition.^24^Figures 6c and S8a present the Raman spectra recorded from the dark-adapted and light-exposed samples of the prepared retina. The differences observed between those spectra can be assigned to the xanthophyll *trans*-to-*cis* photoisomerization, in particular, based on the increase in the intensity of the maximum at 1135 cm^–1^ diagnostic for *cis* molecular configuration forms of carotenoids (see also Figures S4 and S5). Owing to the spectral shift toward higher wavenumbers in the spectra of Lut versus Zea and in the spectra of the 13-*cis* versus all-*trans* configurations one can estimate a distribution of the 13-*cis* forms of Lut in the macula, giving rise to the most blue-shifted band, based on the deconvolution of the original resonance Raman spectra (Figure S8b). Such a distribution map (Figure 6d) shows that the *cis* forms are mainly located in the central macular region characterized by a particularly high concentration of xanthophylls.

## Discussion

**Figure 7:**
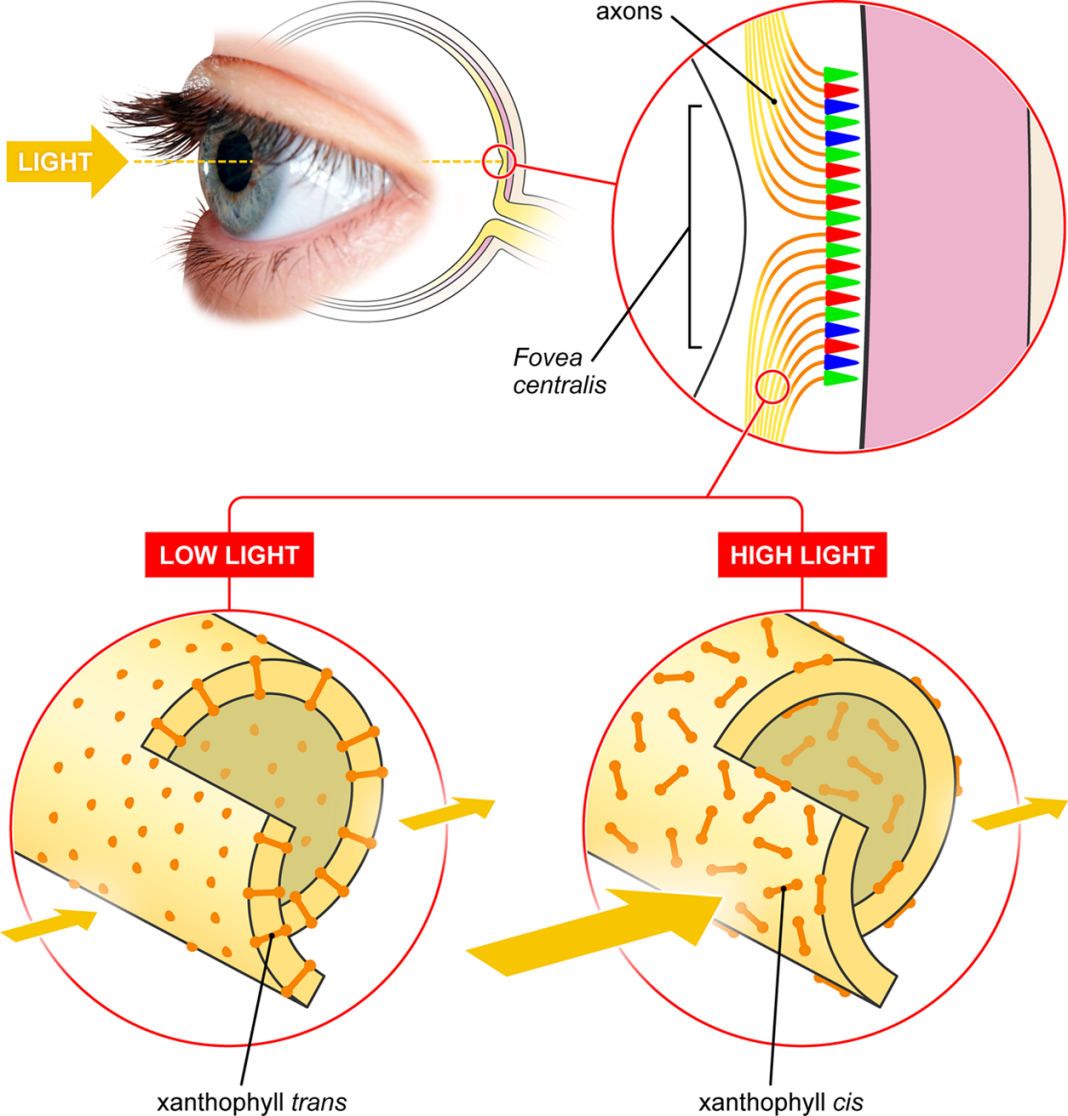
Model of operation of the molecular blinds mechanism. For simplicity, the axons are represented by continuous lines. A single axon oriented in the retina plane is shown magnified, to visualize light-driven xanthophyll reorientation in the membranes.

The results presented here show the activity of molecular mechanisms triggered photochemically and related to each other by a cause-and-effect relationship. The sequence starts with the photoisomerization of the membrane-bound xanthophylls and results in the pigment reorientation leading to a photochromic-type response. Importantly, the operation of the same molecular mechanisms has been identified in this study in model systems, computational studies, and human retina samples, both using FLIM and Raman imaging. Moreover, the interpretations of the spectroscopic effects were confirmed by the results of direct chromatographic analyses. This allows concluding that the mechanism unveiled is universal and can be important from a physiological point of view, playing a regulatory role in the human retina. According to the results of the experiments, such regulation consists in controlling the intensity of light reaching photoreceptors by modulating its absorption in xanthophyll-rich regions of the retina: blocking light transmittance at high intensities and increasing light transmittance at low intensities. This mechanism was found to be particularly active in the central retina (Figure 6d), where the xanthophyll concentration is about 100 times higher than in the peripheral region.^1,2,7^ The exceptionally high concentration of xanthophylls in this region of the retina corresponds to a high risk of photodamage since the *macula lutea* is located on the optical axis of the eye and therefore particularly exposed to a high number of photons. Besides, retinal tissues are highly vascularized, resulting in relatively high molecular oxygen partial pressure, which further increases the risk of photooxidative damage. The macular xanthophylls were shown to be potent antioxidants,^25^ protecting membranes through combined activity of different molecular mechanisms: physical quenching of singlet oxygen, modification of structural and dynamic properties of lipid bilayers, and sacrificial chemical reactions leading to the pigment oxidation.^8,26−29^ An important aspect of the photoprotective activity of xanthophylls in the *macula lutea* is the ability to filter out the short-wavelength radiation due to the high molecular extinction coefficients of this group of pigments.^1,6^ The most plausible physiological role of this mechanism is to enable color and high-acuity vision at dim light while protecting photoreceptors against photodamage at high light. Using an analogy to the macro-scale, we like to refer to this mechanism as “molecular blinds” (Figure 7). Dynamic protection of photoreceptors in the *macula lutea* seems to be particularly important from a physiological point of view. As can be seen from Figure 2c the amplitude of the short-lifetime component changes in the range ca. 0.1–0.7. The maximum light attenuation effect of macular xanthophylls is determined by the maximum optical density of macular pigments, reported being at an average level of ∼0.4,^30−32^ which translates into a factor of 2.5. Changes in the amplitude of the short-lifetime component of xanthophyll fluorescence reported in the present study reflect the range of changes attributed to the xanthophyll photoisomerization process. The fact that this amplitude increases by a factor of 7 can be interpreted that the level of light absorption (1 minus Transmission) increases by the same factor at the fluorescence excitation wavelength. This implies that the optical density due to such an effect can potentially vary within the range ca. 0.04–0.4. One can compare this effect with a physiological regulatory mechanism based on the contraction of the pupil. Narrowing of the human pupil from 7 to 2 mm (see ref (33)) causes a 12-fold reduction in the photon flux arriving at the retina. On the other hand, the pupil contraction mechanism does not substantially protect the central retina (the diameter of ∼2 mm). The fact that this fragment of the retina is especially enriched in xanthophylls^1^ creates unique conditions for the use of these pigments for photoprotection by dynamically regulating the intensity of light reaching the photoreceptors. Importantly, unlike nervous system-controlled processes such as pupil contraction (the reaction time > 0.5 s),^34^ the regulatory mechanism disclosed and characterized in this study responds much faster as it is essentially based on xanthophyll photochemical reactions and molecular dynamics in the membranes (the effect was observed on the timescale < 0.6 × 10^–3^ s, defined by the time of scanning a single voxel in FLIM experiments, Figure 2a). As reported above, the longer exposure to high light gives rise to fluctuations in light absorption by xanthophylls, manifested by fluctuations in fluorescence emission. In our opinion, this effect is associated, most probably, with multiple acts of *trans*–*cis* and *cis*–*trans* photoisomerization (Figure 2e). The reactions in both directions were observed (Figure S6) and can be readily light-driven owing to the relatively low energy barrier for both the *trans*-to*-cis* and *cis*-to-*trans* isomerization.^22^ It means that this type of regulation does not require any particular reversibility since the proportion between the *cis* and *trans* xanthophyll forms is essentially controlled by the actual light intensity. All imaging experiments in our research started at extremely low intensities of laser light that are barely detectable by both the experimental systems and the human eye (∼10^–4^ μW, see Figure 2). Nevertheless, a sharp increase in the amplitude of the short-lifetime component attributed to xanthophylls and interpreted in terms of pigment photoisomerization can be observed at ∼3 × 10^–2^ μW. Providing that the pixel size is 5.6 × 10^–10^ cm^2^ and the scanning time of a single pixel is 0.6 × 10^–3^ s one can estimate the energy dose deposited at a level of 0.03 J/cm^2^. This value corresponds to the 1 s exposition at the absorbed retinal irradiance from the range characterized as a “blue light hazard”.^35^

It has to be noted that a certain fraction of macular xanthophylls is associated in the retina with specific proteins (GSTP1 and StARD3).^36−38^ The results of our experiments show that the light-induced reorientation of xanthophylls in consequence of photoisomerization takes place in the environment of lipid membranes. On the other hand, it is highly probable that light-induced isomerization also occurs in the case of protein-bound pigments. One possible scenario is that after molecular reconfiguration, xanthophylls reversibly detach from the protein due to steric mismatch and locate themselves in the lipid phase of the membrane. The operation of a similar process in the case of retinal is a key mechanism in the visual cycle.^39^ We would like to point out that a similar process has also been reported for the xanthophyll violaxanthin bound to the photosynthetic antenna protein LHCII.^40^ On the other hand, it may not be excluded that the protein-bound fraction of macular xanthophylls is not involved in the mechanism manifested by the photoselection experiments. In our opinion, the solution to this interesting problem deserves specially targeted research.

## Conclusions

We present the operation of a light-intensity-controlled molecular system capable of regulating the transmission of light through the xanthophyll-rich layers of the human retina. This regulatory system, which we call the molecular blinds, is based on the *trans*–*cis* photoisomerization of xanthophylls. It is also a remarkable example of how very similar molecular mechanisms are used in natural systems to perform fundamentally different physiological responses. On the one hand, the *cis*–*trans* photoisomerization of the polyene chromophore in rhodopsin triggers a cascade of signals in photoreceptors.^39^ On the other hand, very similar light-controlled isomerization of xanthophylls in the retina is a process underlying dynamic regulation of the intensity of light transmitted to photoreceptors, thus enabling vision at a very low light intensity and protecting the retina from photodamage when suddenly exposed to strong light.

## Supporting Information Available

Fluorescence analysis of human retina, reversibility of the light-induced fluorescence lifetime changes of axon-bound xanthophylls, fluorescence microscopy analysis of a single lipid vesicle containing xanthophylls, Raman analysis of a single xanthophyll-containing lipid vesicle, resonance Raman analysis of xanthophylls, fluorescence microscopy analysis of single lipid vesicles containing xanthophylls in molecular configuration *cis*, computational analysis of zeaxanthin in the lipid membranes, resonance Raman spectra of the retina, and hydrophobic lengths, defined as the distance between the hydroxyl oxygen atoms (PDF)
