# Supplementary material for: Light-Modulated Sunscreen Mechanism in the Retina of the Human Eye
Source: J Phys Chem B. 2021 May 26;125(23):6090–102. doi: 10.1021/acs.jpcb.1c01198 (PMC8279541; doi:10.1021/acs.jpcb.1c01198)

Supporting Information for Publication

Light-Modulated Sunscreen Mechanism in the Retina of the Human Eye

Rafal Luchowski[1,a], Wojciech Grudzinski[1,a], Renata Welc[1], Maria Manuela Mendes Pinto[1], Alicja Sek[1,2,b], Jan Ostrowski[3,b], Lukasz Nierzwicki[4], Pawel Chodnicki[4], Milosz Wieczor[4], Karol Sowinski[1], Robert Rejdak[3], Anselm G. M. Juenemann[5], Grzegorz Teresinski[6], Jacek Czub[4] and Wieslaw I. Gruszecki[1]*

1. Department of Biophysics, Institute of Physics, Maria Curie-Sklodowska University, Pl. M. Curie-Sklodowskiej 1, 20-031 Lublin, Poland
2. Department of Interfacial Phenomena, Institute of Chemical Sciences, Faculty of Chemistry, Maria Curie-Sklodowska University, Pl. M. Curie-Sklodowskiej 3, 20-031 Lublin, Poland
3. Department of General Ophthalmology, Medical University of Lublin, Chmielna 1, 20-079 Lublin, Poland
4. Department of Physical Chemistry, Gdansk University of Technology, Narutowicza 11/12, 80-233 Gdansk, Poland
5. Viselle Augenzentrum Erlangen GmbH, Erlangen, Germany
6. Department of Forensic Medicine, Medical University of Lublin, Jaczewskiego 8b, 20-090 Lublin, Poland

a - these authors contributed equally to this work

b - these authors contributed equally to this work

* - corresponding author Email: wieslaw.gruszecki@umcs.pl (ORCID 0000-0002-8245-3913)

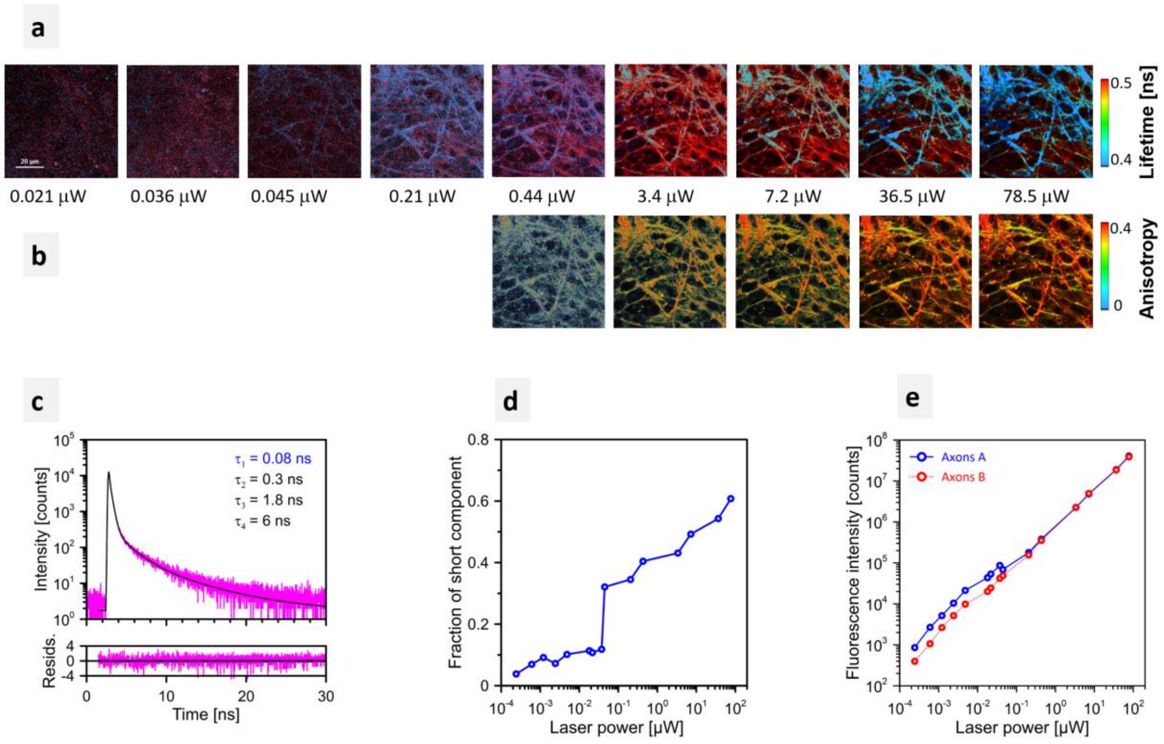

Figure S1. Fluorescence analysis of the human retina. (**a**) FLIM imaging with increasing laser power. The retina from a healthy 18-year-old female donor. (**b**) Fluorescence anisotropy-based images corresponding to the FLIM images presented in panel a. Due to the very low number of detected photons at very low laser powers it was not able to determine reliable fluorescence anisotropy levels. Note the high fluorescence anisotropy values corresponding to the higher light doses. Such an effect is consistent with the orientation of a substantial number of fluorophores parallel to the electric vector of the excitation light beam, in the plane of scanning (the plane of the retina). (**c**) Fluorescence decay kinetics recorded from axons. Fluorescence photons were collected with the FLIM confocal microscopy system focused in the outer plexiform layer of the central retina. Excitation with a 470 nm laser. The black curve represents the re-convolution dependency based on the four-exponential fit. A quality of the fit, represented by residuals, is presented in the lower part of the graph. The inset shows fluorescence lifetime components. The component $\tau = 0.080 \pm 0.005$ ns represents the macular xanthophylls. The retina from a healthy 57-year-old male donor. (**d**) Laser power dependency of the fraction of the short lifetime component in the experiment presented in panel a. (**e**) Total number of detected fluorescence photons in a function of the excitation laser power. The dependencies represent the imaging experiments shown in Fig. 2a and analyzed in Fig. 2c (Axons A) and in Fig. S1a (Axons B).

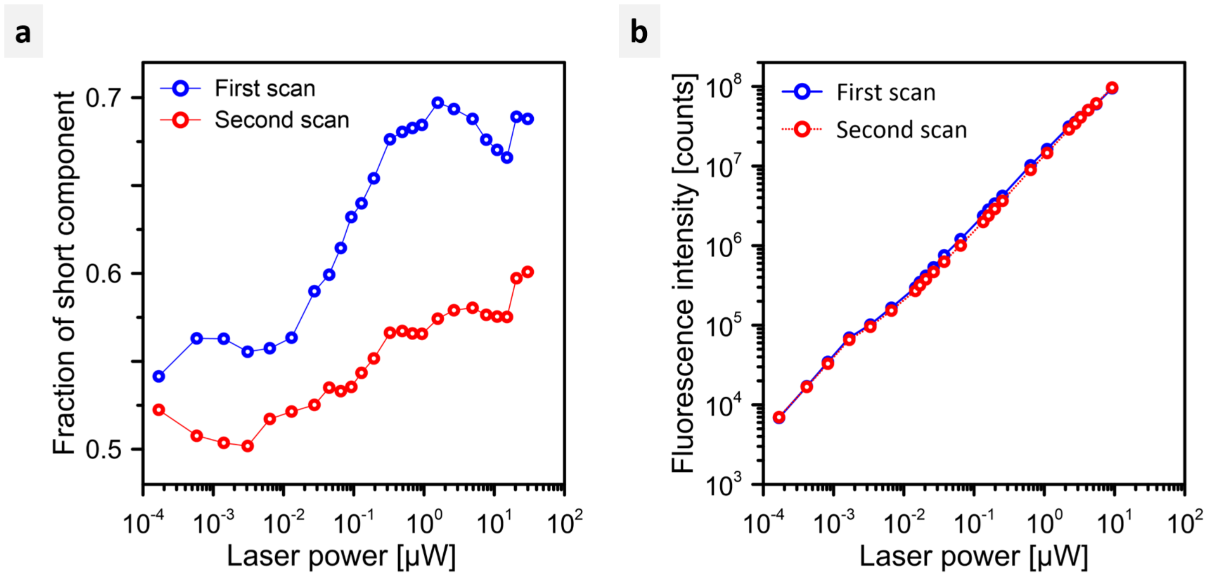

Figure S2. Reversibility of the light-induced fluorescence lifetime changes of axon-bound xanthophylls. (**a**) Dependence of an amplitude of the short-lifetime component representing xanthophylls on scanning laser power. The dependencies correspond to the experiments of axon imaging (as in the case of Fig. 2a and Fig. S1a) repeated twice from the same area after the 15 min period of dark adaptation of the sample. The initial fraction level of the short lifetime component and range of changes varies depending on the location in the retina. Such a dependency can be explained in terms of different proportions of Zea plus *m*-Zea and Lut, given that only Lut is subjected to light-induced isomerization and reorientation. (**b**) Total number of fluorescence photons in a function of the excitation laser power recorded in the experiments presented in panel a. The retina from a healthy 37-year-old male donor.

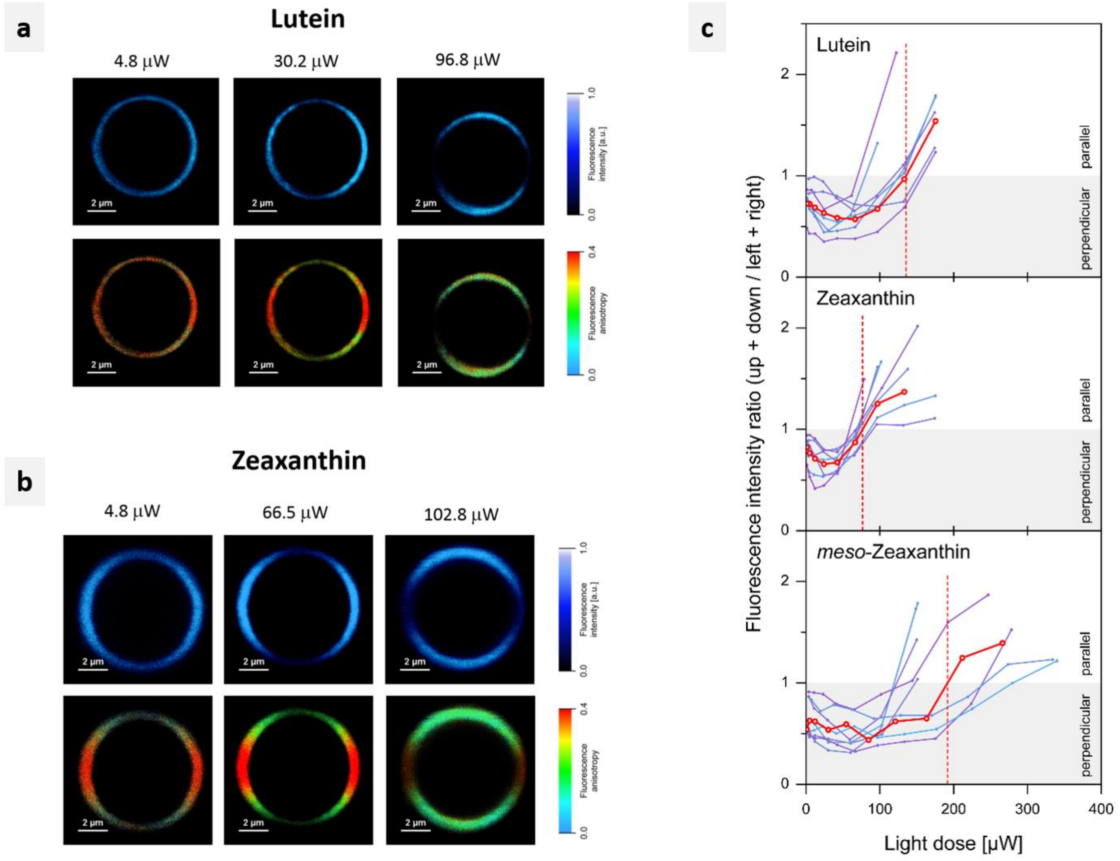

Figure S3. Fluorescence microscopy analysis of a single lipid vesicle containing xanthophylls. (**a**) Confocal fluorescence and fluorescence anisotropy images of the equatorial cross-section of the same lipid vesicle formed with DPPC and containing all-*trans* Lut at a concentration of 0.5 mol% with respect to lipid. Images were recorded at different laser light dose (cumulative power, indicated). (**b**) Caption the same as in panel a, except that the lipid vesicle contained 0.5 mol% of all-*trans* Zea. (**c**) Analysis of fluorescence distribution in xanthophyll-containing GUV. Fluorescence intensity ratio calculated based on the results of the integration of emission photons in the sectors defined by the dashed lines in Fig. 3a: up+down/left+right versus cumulative power of scanning laser. Due to the fact that repeated scans at constant laser power caused changes in the images in terms of the spatial distribution of signal intensity, the results of experiments consisting of a series of subsequent imaging were presented as a function of the cumulated power of laser light, referred to as the dose of light. Each dependency represents a separate experiment. Simplifying, it can be interpreted that the ratio below 1 represents the tendency of xanthophylls to adopt the orientation perpendicular to the membrane plane while the ratio values higher than 1 represent the tendency of xanthophylls to adopt the horizontal orientation. The red plots represent the arithmetic mean of the experimental dependencies shown. The red dashed lines point the crossing of the average plots with the axes corresponding to the ratio level 1. A more detailed analysis of the dependencies reveals appearance of a local minimum in the low light dose region, indicating that the exposure of the samples to relatively low light intensities prompts xanthophylls to adopt the more vertical orientation and only exposure to strong light shifts the equilibrium towards the horizontal orientation.

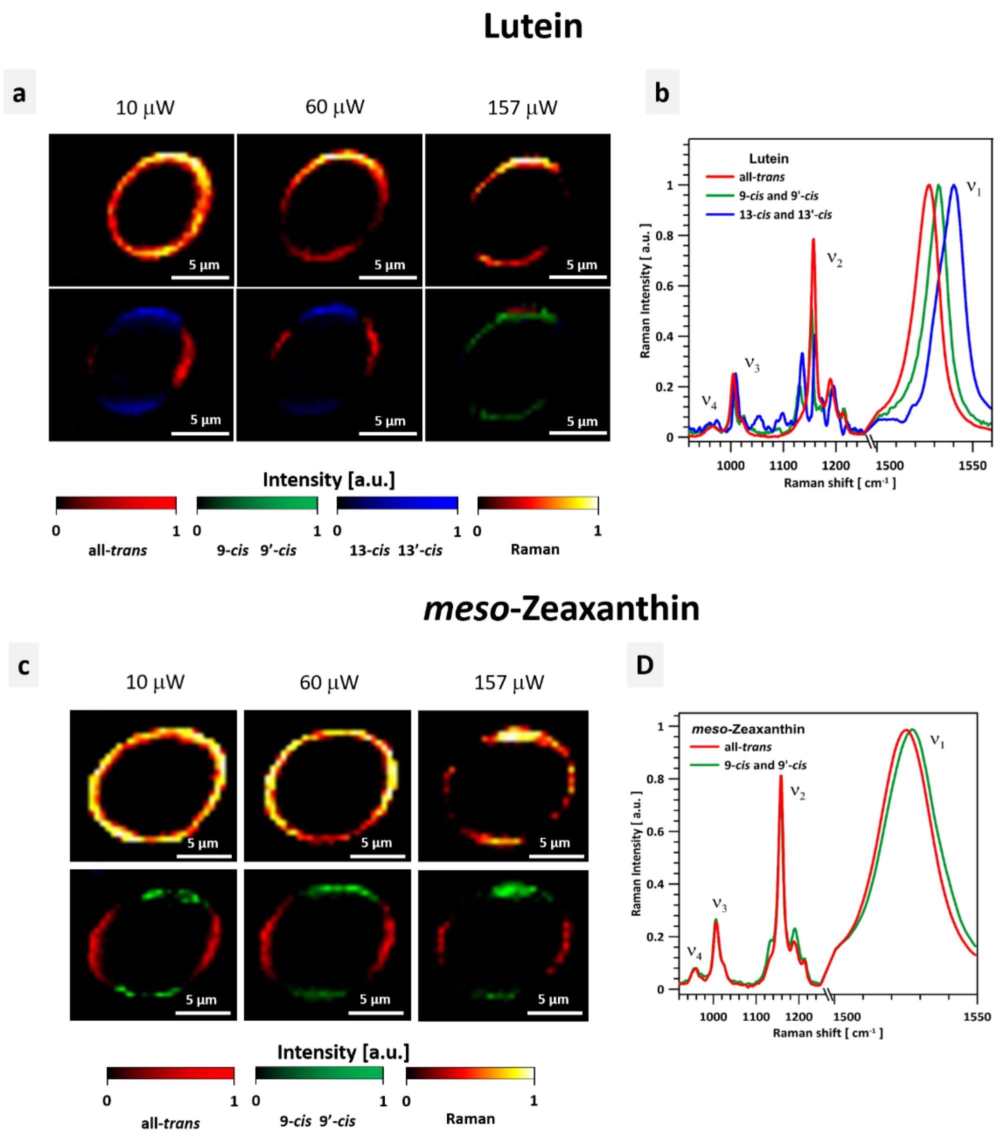

Figure S4. Raman analysis of a single xanthophyll-containing lipid vesicle. (**a**) Confocal resonance Raman images of the equatorial cross-section of the lipid vesicle formed with DPPC and containing all-*trans* Lut at a concentration 0.5 mol% with respect to lipid. Images were recorded at different laser power (cumulative power indicated). The bottom panel shows the results of the principal component analysis. Three components have been resolved, representing Lut molecular configurations: all-*trans*, the mixture of 9-*cis* and 9'-*cis* and the mixture of 13-*cis* and 13'-*cis*, based on the resonance Raman spectra displayed in panel (**b**). (**c**) Caption the same as in panel a except that 0.5 mol% of all-*trans* *m*-Zea was present instead of Lut. Two components have been resolved, representing *m*-Zea molecular configurations: all-*trans* and the mixture of 9-*cis* and 9'-*cis*, based on the resonance Raman spectra displayed in panel (**d**). The assignment of the components is based on the literature (A. Sek et al., Raman spectroscopy analysis of molecular configuration forms of the macular xanthophylls. J. Raman Spectr. 1-7, DOI: 10.1002/jrs.5818 (2020)).

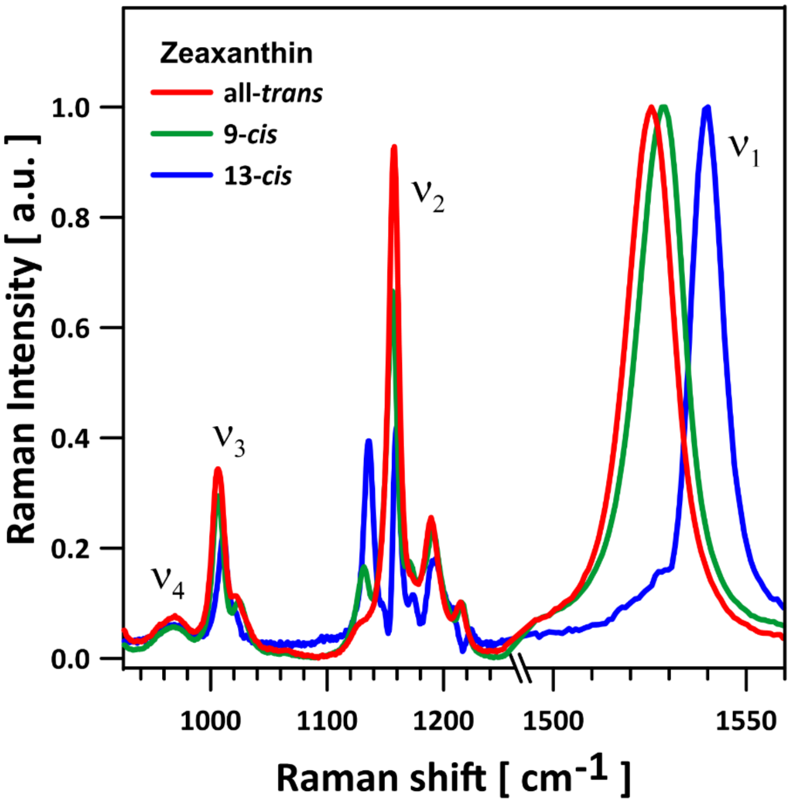

Figure S5. Resonance Raman analysis of xanthophylls. Spectral components identified in the imaging of Zea-containing GUV (shown in Fig. 3b) The assignment and deconvolution based on the approach described in detail previously (A. Sek et al., Raman spectroscopy analysis of molecular configuration forms of the macular xanthophylls. J. Raman Spectr. 1-7, DOI: 10.1002/jrs.5818 (2020)).

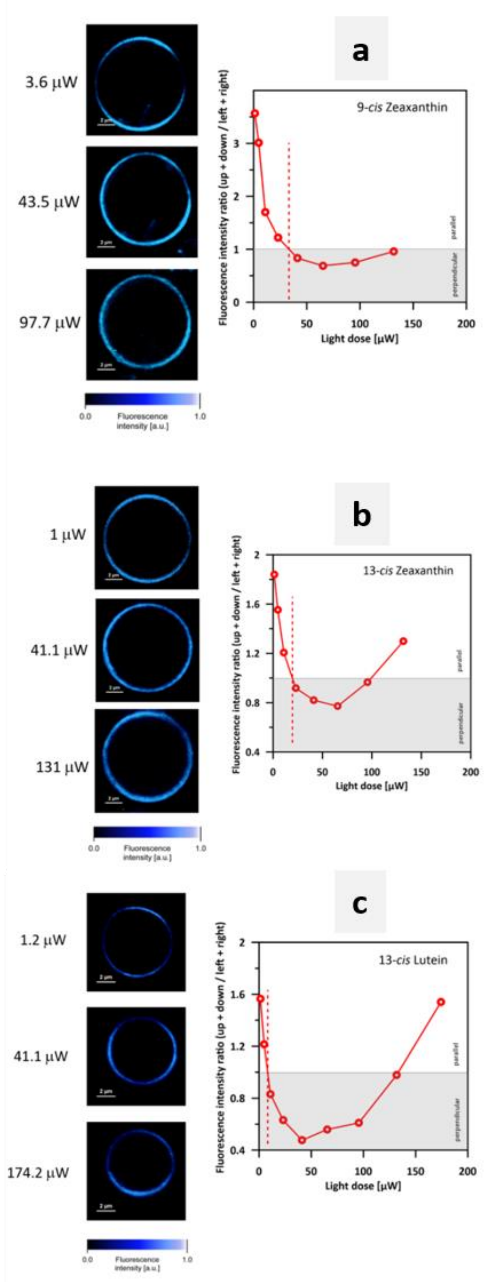

Figure S6. Fluorescence microscopy analysis of single lipid vesicles containing xanthophylls in molecular configuration *cis*. (**a**) Confocal images of the equatorial cross-section of the same lipid vesicle formed with DPPC and containing 9-*cis* Zea at a concentration of 0.5 mol% with respect to lipid. Images were recorded at different laser light power (cumulative power referred to as light dose indicated, see the explanation in the caption to Figure S3c). The full series of images is analyzed quantitatively in the right-hand panel, based on the methodology explained in Fig. 3 and Fig. S3c. (**b**) Caption as in panel a, except that 13-*cis* Zea was incorporated. (**c**) Caption as in panel a, except that 13-*cis* Lut was incorporated. As can be seen, the increase in light intensity results in the light-induced reorientation of xanthophyll molecules. This effect can be explained in terms of a *cis*-*trans* photo-conversion of xanthophylls. The net yield of *trans*-*cis* and *cis*-*trans* reactions at a given light intensity is directly responsible for the number of molecules in each of the isomer fractions, and consequently for the dominant orientation of xanthophylls in membranes.

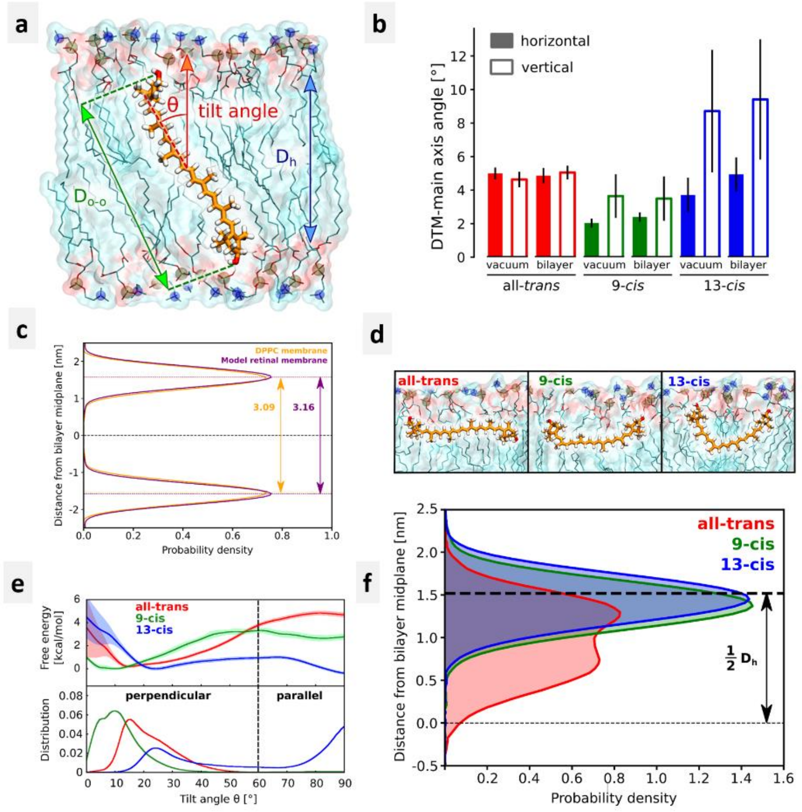

Figure S7. Computational analysis of Zea in the lipid membranes. (**a**) Definitions of the geometric parameters used in the article. Tilt angle θ was defined as the angle between the vector connecting the centers of mass of the carbon atoms of each half of the polyene chain (C8-C15) and the bilayer normal. The hydrophobic length of xanthophylls $D_{O\text{-}O}$ was computed as the distance between their hydroxyl oxygen atoms. The membrane hydrophobic thickness D_h was defined as the distance between the average positions of the carbonyl carbon atoms (C1) in the opposite leaflets. (**b**) Mean angles between the transition dipole moment vector and the main axis of the xanthophyll molecule. Angles were calculated on the ONIOM/TD-DFT/MN12L level for three isomers: all-trans, 9-*cis* and 13-*cis* Zea. Transition dipole moments were calculated in both membrane orientations - horizontal and vertical - and the effect of the charge distribution of the environment was assessed by repeating the calculations in vacuum. Each mean was calculated based on 10 representative structures extracted from classical MD simulations. (**c**) Analysis of the thickness of the hydrophobic core of the lipid bilayer. Distributions of the position of the carbonyl carbon atoms (C1) along the bilayer normal for the DPPC (orange) and model retinal (magenta) membranes. Arrows show the average hydrophobic thickness D_h of both bilayers in nm. (**d**) Representative structures of the three considered Zea isomers oriented parallel to the surface of the DPPC membrane. (**e**) Free energy profiles (top) and the corresponding probability densities (bottom) for the tilt angle between the polyene chain and the membrane normal (see panel a) for Zea all-*trans* and its 9-*cis* and 13-*cis* isomers in the model retinal membrane. (**f**) Probability distribution of the position of the Zea hydroxyl groups along the bilayer normal for the fraction of parallel-oriented Zea molecules (θ > 60°). The hydrophobic thickness of a single leaflet of the DPPC membrane is shown as a thick dashed line.

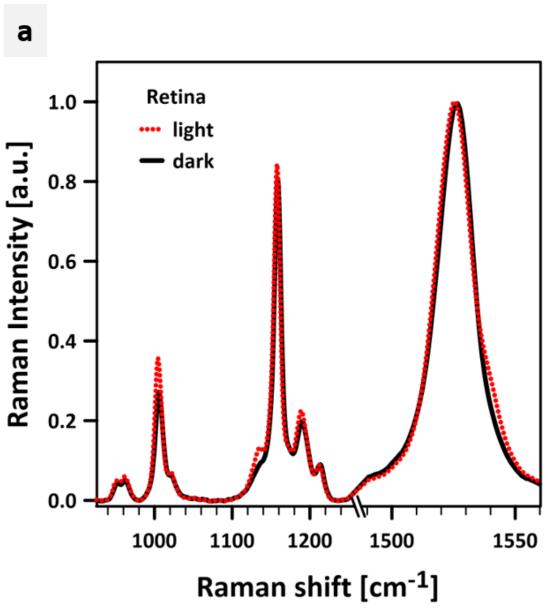

Figure S8. Resonance Raman spectra of the retina. Raman spectra recorded with the laser line 488 nm, at -77°C. The retina from a healthy 34-year-old female donor. (**a**) The spectra were recorded from the adjacent regions of the central retina, dark-adapted and illuminated for 5 min with white light (750 µmol photons m^{-2} s^{-1}). The spectra are normalized at the maximum. A component analysis (spectral deconvolution) of the "light" spectrum is shown in panel (**b**). Three spectral forms have been resolved assigned to the Lut molecular configurations: all-*trans*, a mixture of 9-*cs* and 9'-*cis* and a mixture of 13-*cis* and 13'-*cis* (marked). The assignment and deconvolution based on the approach described in detail previously (A. Sek et al., Raman spectroscopy analysis of molecular configuration forms of the macular xanthophylls. J. Raman Spectr. 1-7, DOI: 10.1002/jrs.5818 (2020)).

Table S1. Hydrophobic lengths, defined as the distance between the hydroxyl oxygen atoms (Figure S8A), of all possible stable conformers of the studied xanthophylls in their structures geometry-optimized at the MN12-SX/6-31G(d) level of theory. The conformers are labeled as in the scheme below presenting the structural representations of the β-ionone (left) and ε-ionone (right) rings of the xanthophyll molecules. Corresponding potential energy profiles for the rotation of the selected dihedral angles are also shown (highlighted in blue) describing the orientation of the two ring types with respect to the polyene chain.

	$D_{O\text{-}O}$ [Å]					
Xanthophyll	all-*trans*	9-*cis*	13-*cis*	15-*cis*	13'-*cis*	9'-*cis*
**Zeaxanthin**						
I-I	29.74	26.00	21.98	19.34		
I-II	28.10	23.09	19.16	18.46		
I-III	28.93	24.99	20.19	19.24		
II-II	28.40	24.08	19.34	19.06		
II-III	29.18	25.37	21.17	20.44		
III-III	29.55	25.81	21.52	20.00		
**Lutein**						
I-1	30.70	28.32	23.76	22.52	23.06	27.66
II-1	29.33	25.31	21.56	19.80	20.36	24.85
III-1	29.93	25.63	22.74	20.58	21.77	26.44
I-2	28.92	25.40	20.40	18.95	20.19	24.84
II-2	27.56	22.63	18.71	16.42	17.14	21.82
III-2	28.23	23.90	19.38	18.57	18.32	23.74
**meso-Zeaxanthin**						
I-I	30.45	26.37	20.93	19.35	21.41	25.99
I-II	30.24	24.74	20.55	18.85	20.42	25.65
I-III	30.59	26.52	21.42	20.65	21.22	26.21
II-II	30.42	25.99	21.41	19.43	20.93	26.13
II-III	30.77	26.26	22.26	20.29	21.63	26.86
III-III	31.10	27.08	22.16	20.37	22.16	27.08

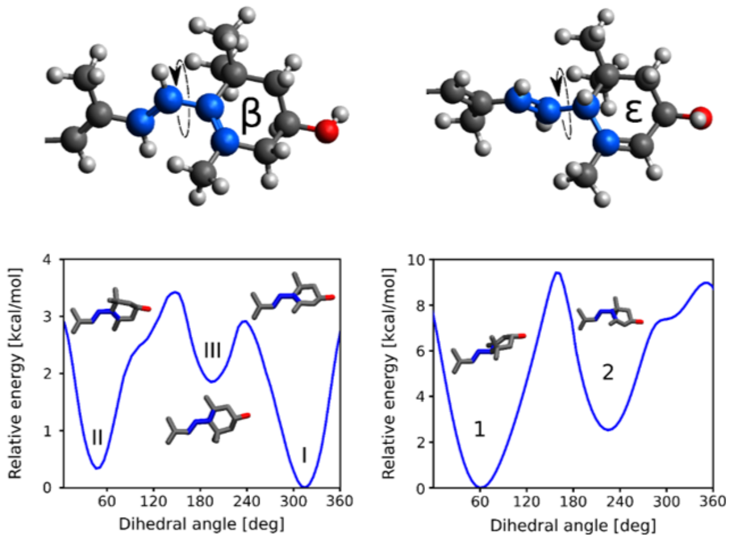

Supplement: Supplementary file 1 — jp1c01198_si_001.pdf [file jp1c01198_si_001.pdf]
